# α,β-Amyrin prevents steatosis and insulin resistance in a high-fat diet-induced mouse model of NAFLD via the AMPK-mTORC1-SREBP1 signaling mechanism

**DOI:** 10.1590/1414-431X2021e11391

**Published:** 2021-08-13

**Authors:** R.P. de Lima, P.I.G. Nunes, A.F.S.C. Viana, F.T.B. de Oliveira, R.A.C. Silva, A.P.N.N. Alves, D.A. Viana, S.G.C. Fonseca, A.A. Carvalho, M.H. Chaves, V.S. Rao, F.A. Santos

**Affiliations:** 1 Departamento de Fisiologia e Farmacologia, Faculdade de Medicina, Universidade Federal do Ceará, Fortaleza, CE, Brasil; 2 Departamento de Clínica Odontológica, Faculdade de Farmácia, Odontologia e Enfermagem, Universidade Federal do Ceará, Fortaleza, CE, Brasil; 3 Laboratório de Patologia e Medicina Legal, Faculdade de Ciência Veterinária, Universidade Estadual do Ceará, Fortaleza, CE, Brasil; 4 Departamento de Farmácia, Faculdade de Farmácia, Odontologia e Enfermagem, Universidade Federal do Ceará, Fortaleza, CE, Brasil; 5 Instituto Federal de Educação, Ciência e Tecnologia do Piauí, Piripiri Campus, Piripiri, PI, Brasil; 6 Departamento de Química, Ministro Petrônio Portella Campus, Universidade Federal do Piauí, Teresina, PI, Brasil

**Keywords:** α,β-Amyrin, High-fat diet, Nonalcoholic fatty liver disease, Hepatosteatosis, Signaling pathways, Lipid metabolism

## Abstract

Nonalcoholic fatty liver disease (NAFLD), characterized by hepatosteatosis and steatohepatitis, is intrinsically related to obesity. Our previous study reported on the anti-obese activity of α,β-amyrin (AMY), a pentacyclic triterpene isolated from *Protium heptaphyllum*. This study investigated its ability to prevent fatty liver and the underlying mechanism using the mouse model of NAFLD. NAFLD was induced in male Swiss mice fed a high fat diet (HFD) for 15 weeks. The controls were fed a normal chow diet (ND). The mice were simultaneously treated with AMY at 10 and 20 mg/kg or fenofibrate at 50 mg/kg. Lipid levels along with metabolic and inflammatory parameters were assessed in liver and serum. The liver sections were histologically examined using H&E staining. RT-qPCR and western blotting assays were performed to analyze signaling mechanisms. Mice fed HFD developed severe hepatic steatosis with elevated triglycerides and lipid droplets compared with ND controls. This was associated with a decrease in AMP-activated protein kinase (AMPK) activity, an increase of mechanistic target of rapamycin complex 1 (mTORC1) signaling, and enhanced sterol regulatory element binding protein 1 (SREBP1) expression, which have roles in lipogenesis, inhibition of lipolysis, and inflammatory response. AMY treatment reversed these signaling activities and decreased the severity of hepatic steatosis and inflammatory response, evidenced by serum and liver parameters as well as histological findings. AMY-induced reduction in hepatic steatosis seemed to involve AMPK-mTORC1-SREBP1 signaling pathways, which supported its beneficial role in the prevention and treatment of NAFLD.

## Introduction

Nonalcoholic fatty liver disease (NAFLD) is a spectrum of liver diseases that comprises hepatosteatosis and nonalcoholic steatohepatitis (NASH), progressing to fibrosis and cirrhosis (1,2). Hepatic steatosis is characterized by the accumulation of free fatty acids and cholesterol in liver tissues, whereas NASH is associated with hepatocyte death, inflammation, and fibrosis (3). The global prevalence of NAFLD in the general population has been estimated at 25% and the prevalence rate in South America is higher than that reported for the US, approximately 30-45% (4). The persistent increase in liver lipids in NAFLD, such as diacylglycerols and triglycerides, intensifies hepatic insulin resistance, which may further lead to cardiovascular complications and type 2 diabetes (5). Currently, there is no approved pharmacological treatment for NAFLD (6), so a need exists to develop drugs that are effective against NAFLD and the associated health risks (7). In this regard, there is growing interest in the search for natural medicinal substances of plant origin that inhibit fatty liver (8,9).

α,β-Amyrin (AMY) is a pentacyclic triterpenoid isolated from *Protium heptaphyllum* (Aubl.) March (10) that has anti-inflammatory (11), hepatoprotective (12), anti-hyperglycemic, and hypolipidemic (13) properties. Previously, we reported the anti-obesity effect of AMY in high-fat diet (HFD)-fed mice (14), and more recently its anti-adipogenic effect via modulation of lipid and carbohydrate metabolism in 3T3-L1cells (15). Based on these studies, we hypothesized that AMY might prevent NAFLD by regulating the abnormal lipid metabolism in the liver. HFD-fed animals exhibit obesity, insulin resistance (IR), and dyslipidemia, conditions common to the development of NAFLD (6,16). Therefore, we selected the HFD-fed mouse model to investigate the efficacy of AMY for the prevention of NAFLD, and further aimed to establish the underlying molecular mechanism, analyzing AMP-activated protein kinase (AMPK), the mechanistic target of rapamycin complex 1 (mTORC1), and the sterol regulatory element binding protein 1 (SREBP1) transcriptions, which all have a pivotal role in hepatic lipid homeostasis ([Bibr B17]-[Bibr B18] [Bibr B19] 20).

## Material and Methods

### Extraction and isolation of α- and β-amyrin

**Figure 1:**
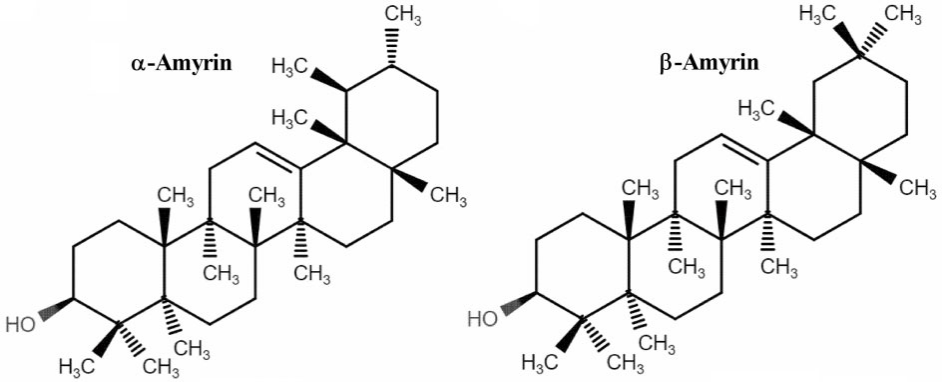
Chemical structure of α-amyrin (A) and β-amyrin (B).

The extraction and isolation of α- and β-amyrin (Figure 1) from the crude resin obtained from trunk wood of *Protium heptaphyllum* was carried out as described earlier (10) and its structural identity was confirmed by ^1^H and ^13^C NMR spectral analysis, based on the method developed by Olea and Roque (21), and based on literature data (22). The ratio of α- and β-amyrin in this mixture was 63:37, calculated by ^1^H NMR, by dividing the signal area of olefinic hydrogens δ=5.14 (α-amyrin) and δ=5.20 (β-amyrin) by the signal area in δ=3.24 (dd, J=11.0 and 5.0 Hz), attributed to H-3 in the two triterpenes, and multiplied by 100. The optical rotation for the mixture was ${\left[\alpha \right]}_{D}^{20}$ +92.5 (*c* 0.5 in CHCl_3_).

### Animals and experimental groups

Male Swiss mice weighing 20-25 g obtained from the Central Animal House of the Federal University of Ceará were used. They were housed in a controlled environment (24±2°C, 55±5% relative humidity, 12-h light/dark cycle) with food (chow) and water provided *ad libitum* unless otherwise noted. All experiments involving the mice were approved by the Institutional Animal Care and Use Committee of Federal University of Ceará (Protocol Number 5347120318), in accordance with the Guidelines of the National Institutes of Health.

For experimentation, mice were fed either a normal diet (ND) or a high-fat diet (HFD) for 15 weeks, after which lipid levels and metabolic and inflammatory parameters were assessed in livers and serum of the mice. After a one-week adaptation, the mice were sorted into five treatment groups as follows (n=8/group): normal diet (ND); high-fat diet (HFD); HFD + α,β-amyrin (AMY, 0.05% in drinking water, which is equivalent to a dose of 10 mg/kg based on water consumption); HFD + AMY (0.1% in drinking water, equivalent to 20 mg/kg); or HFD + fenofibrate (FEN, 0.25% in drinking water, equivalent to 50 mg/kg; Lipidil^®^, Abbott, USA) for 15 weeks. FEN, a known hypolipidemic drug, was used as control for comparison (23). Laboratory pellet chow was used as the control diet (Nuvilab, Brazil), consisting of 19% proteins, 56% carbohydrates, 3.5% lipids, 4.5% cellulose, 5% vitamins and minerals, and 12% humidity. The standardized HFD used for the study (24) contained 20% proteins, 48% carbohydrates, 20% lipids, 4% cellulose, 5% vitamins and minerals, and 3% humidity. The choice of the drug concentrations adopted for AMY and FEN was based on preliminary studies that showed their safety and efficacy (14,25). AMY and FEN were suspended initially in 2% (v/v) Tween 80 and then further in water. HFD-fed controls received the same vehicle. AMY or vehicle was changed twice a week. The body weight of each mouse was measured weekly, as were the consumption of food (g) and water (mL). At the end of the experimental period, the animals were starved for 6 h, and blood was taken by venous puncture under light anesthesia with diethyl ether. Blood samples were centrifuged at 2000 *g* for 10 min at 4°C. The serum was then separated and used within a few hours or frozen at -80°C until analysis. The liver and visceral adipose tissue were removed, weighed, and expressed in milligrams per 10 g body weight.

### Glucose and insulin tolerance tests

Glucose tolerance test (GTT) and insulin tolerance test (ITT) were performed, respectively, to investigate glucose and insulin sensitivity in the HFD-fed mice treated or not with test drugs, during the 13th and 14th weeks of the experiment. For GTT, mice were fasted for 6 h and treated intraperitoneally with 2 g/kg of glucose (Dinâmica, Brazil) in 0.9% saline. Tail-vein blood samples were obtained and blood glucose was determined at times 0 (basal level), 15, 30, 60, 90, and 120 min after glucose injection using an automatic FreeStyle Optium Neo glucometer (Abbott). For ITT, mice were fasted for 4 h and treated intraperitoneally with 0.75 U/kg of human insulin (Eli Lilly, USA) in 0.9% saline. Blood glucose was determined as described for GTT. The area under the curve (AUC) was calculated for both tests (26).

### Serum biochemical analysis

The serum levels of glucose, triglycerides (TG), total cholesterol (TC), high-density lipoprotein cholesterol (HDL-c), very-low-density lipoprotein (VLDL-c), low-density lipoprotein cholesterol (LDL-c), and activities of aspartate aminotransferase (AST) and alanine aminotransferase (ALT) were determined by enzymatic colorimetric assays (Labtest Diagnóstica, Brazil) according to the manufacturer's instructions. Serum insulin was measured by ELISA (Millipore, USA). The homeostasis model assessment of insulin resistance (HOMA-IR index) was calculated as [fasting insulin concentration (mU/L) × fasting glucose concentration (mM)] / 22.5.

### Hepatic lipid extraction and measurement

Hepatic lipids were extracted using the Folch method (27). Briefly, liver tissue was homogenized with chloroform:methanol (2:1, v/v). The homogenate was vortexed and centrifuged (2000 *g* at 25°C for 20 min), the chloroform layer was dried, and the residue was dissolved in isopropanol/Triton X-100 (10%). Hepatic triglycerides (TG) and total cholesterol (TC) concentrations were determined using commercial kits (Labtest Diagnóstica).

### Hepatic glycogen determination

Liver glycogen content was assayed by the anthrone reagent method (28). The liver tissue was mixed with 30% KOH, heated to 100°C for 30 min, and 95% ethanol was added. The pellet was mixed with distilled water and then 0.2% anthrone (in 95% H_2_SO_4_) was added, and absorbance was measured at 620 nm. The results were calculated based on a standard calibration curve for glucose.

### Histological analysis of liver tissue

Liver tissues were excised, fixed in 10% formalin, and processed routinely for paraffin embedding. Tissue sections (4 μm thick) were cut, processed with hematoxylin and eosin staining, and examined under a light microscope. Histological variables were blindly semi-quantitated from 0 to 3 with respect to: steatosis (0: <5% hepatocytes involved; 1: 5-33%; 2: >33-66%; 3: >66%), lobular inflammation (0: no foci; 1: <2 foci per 200× field; 2: 2-4 foci per 200× field; 3: >4 foci per 200× field), and ballooning (0: none; 1: few balloon cells; 2: many cells/prominent ballooning) (29).

### RT-qPCR analysis

Reverse transcription-quantitative polymerase chain reaction (RT-qPCR) was used to analyze relative mRNA expression changes of CD36, FAS, and acetyl-CoA carboxylase 1 (ACC1). Total RNA was isolated from the frozen liver tissue using the QIAzol Lysis RNeasy Lipid Tissue Mini kit (Qiagen, Germany) according to the manufacturer's protocol. Then, the quality of total RNA was evaluated using a NanoDrop 2000 spectrophotometer (Thermo Fisher Scientific, USA). The cDNA was synthesized using the High-Capacity cDNA Reverse Transcription kit (Thermo Fisher Scientific). RT-qPCR was performed using GoTaq^®^ kit Master Mix containing a SYBR green^®^ probe (Promega, USA) in an Mx3005p PCR thermocycler system. PCR amplification was performed as follows: pre-degeneration step at 95°C for 2 min, followed by 40 cycles of amplification/degeneration step at 95°C for 15 s, and annealing/extension step at 59°C for 60 s. Relative quantification was calculated using the 2^−ΔΔCT^ method (30). The primers used are shown in Table 1.

**Table 1:** The primer sequences used for the reverse transcription-quantitative polymerase chain reaction.

Genes	Forward primer (5′-3′)	Reverse primer (5′-3′)
CD36	GCTTGCAACTGTCAGCACAT	GCCTTGCTGTAGCCAAGAAC
FAS	CTGAGATCCCAGCACTTCTTGA	GCCTCCGAAGCCAAATGAGTGA
ACC1	CGCTCAGGTCACCAAAAAGAAT	GTCCCGGCCACATAACTGAT
β-Actin	GGGAATGGGTCAGAAGGACTC	GGTGTGGTGCCAGATCTTCTC

CD36: cluster of differentiation 36; FAS: fatty acid synthase; ACC1: acetyl-CoA carboxylase 1.

### Western blot analysis

Liver tissues were homogenized in protein with RIPA lysis buffer supplemented with sodium orthovanadate (200 mM), phenylmethylsulfonyl fluoride (200 mM), and protease inhibitor cocktail, and the protein was extracted as described previously (31). Total protein (15 μg) was separated on 8% SDS-PAGE and transferred onto PVDF membranes (Bio-Rad Laboratories, USA). After blocking with 5% nonfat dry milk in TBST 1x, the membranes were incubated with primary polyclonal antibodies (1:1000), including anti-AMPK (Cell Signaling, USA, cat. No. 2532), anti-pAMPK (Cell Signaling, cat. No. 2531), anti-SREBP1 (Santa Cruz, USA, cat. No. 8984), anti-PPARα (Santa Cruz, cat. No. 9000), anti-mTORC (Cell Signaling, cat. No. 2972), anti-pmTORC1 (Cell Signaling, cat. No. 5536), and anti-β-actin (Cell Signaling, cat. No. 4967) overnight at 4°C. The incubation with the HRP-linked secondary antibodies (1:3000), IgG anti-rabbit antibody (Cell Signaling, cat. No. 7074), and IgG anti-mouse antibody (Cell Signaling, cat. No. 7076) for chemiluminescent detection was at room temperature for 2 h. β-Actin was used as the loading control. Amersham ECL™ Prime Western blotting detection reagent (Bio-Rad Laboratories) was used. ChemiDoc™ MP Image System with Image Lab™ 5.1 software (Bio-Rad Laboratories) was used for acquisition and analysis of western blot images.

### Statistical analysis

Data are reported as means±SE or as median (maximum-minimum). Statistical analysis was carried out with one-way analysis of variance (ANOVA) followed by the Student*-*Newman*-*Keuls test or two-way ANOVA followed by the Bonferroni's test. The nonparametric data are reported as the median values and were analyzed by the Kruskal-Wallis test followed by Dunn's test. The level of significance was set at P<0.05. Statistical analysis was accomplished by Graph Pad Prism 5.0 (GraphPad Software Inc., USA).

## Results

### AMY prevented HFD-induced metabolic disorder and insulin resistance

Initially, since NAFLD is a hepatic manifestation of metabolic syndrome (i.e., visceral obesity and insulin resistance), we investigated the potential protective effects of AMY and FEN against HFD-induced metabolic disorders and insulin resistance in mice fed the HFD for 15 weeks. As shown in Table 2, initial body weights were not significantly different between groups, but at the end of 15 weeks, significant differences were observed in final body weights and net food consumption based on diet. The mice fed the HFD gained significantly more weight (23.8%) than those fed the ND, and final body weights were significantly lower in mice receiving AMY at 10 and 20 mg/kg (16.3 and 14.1%, respectively) or FEN (18.2%), compared to the HFD control. Those fed the HFD consumed significantly more food (14.3%) than those fed the ND, but not if given AMY or FEN. The differences in water consumption among the groups were not statistically significant. The relative weight of accumulated visceral adipose tissue in mice fed the HFD was significantly higher (688.5%) than in those fed the ND. However, the animals treated with AMY or FEN accumulated significantly less visceral adipose tissue (Table 2).

**Table 2:** Effect of α,β-amyrin (AMY) and fenofibrate (FEN) treatments on body weight changes, food and water intake, serum glucose and insulin levels, and insulin resistance in mice fed the high-fat diet (HFD) or normal diet (ND) for 15 weeks.

Parameters	ND	HFD	HFD+AMY (10 mg/kg)	HFD+AMY (20 mg/kg)	HFD+FEN (50 mg/kg)
Initial body weight (g)	24.85±0.18	25.56±0.34	24.72±0.23	24.93±0.33	24.60±0.90
Final body weight (g)	46.22±0.72	57.22±1.44^#^	47.88±0.98*	49.14±2.63*	46.82±1.70*
Food intake (g/week)	44.95±1.08	51.37±2.68^#^	37.48±1.41*	39.47±1.92*	42.12±1.33*
Water intake (mL/week)	66.70±1.67	62.28±2.06	63.67±2.45	68.56±4.56	60.35±1.45
Visceral adipose tissue (mg/10 g)	92.47±29.35	729.10±20.50^#^	442.00±32.84*	412.40±40.81*	235.20±43.42*
Glucose (mg/dL)	91.71±8.69	174.3±13.21^#^	83.67±4.91*	100.50±9.80*	98.89±14.34*
Insulin (ng/mL)	1.50±0.21	4.52±0.60^#^	2.16±0.55*	1.91±0.36*	1.73±0.40*
HOMA-IR	8.25±1.63	46.30±5.67^#^	10.38±2.74*	12.04±2.36*	7.52±2.37*

Data are reported as means±SE (n=8); ^#^P<0.05 *vs* ND-fed animals; *P<0.05 *vs* HFD-fed animals (one-way ANOVA followed by Student Newman Keul's multiple comparison *post hoc* test). HOMA-IR: homeostasis model assessment of insulin resistance.

As shown in Table 2, fasting blood glucose and insulin levels in the HFD-fed mice were significantly higher (90 and 201.3%, respectively) compared to the ND fed mice, and consequently the HOMA-IR value was significantly greater in the HFD group, demonstrating the presence of insulin resistance. The animals treated with AMY at 10 and 20 mg/kg or FEN demonstrated a higher degree of insulin sensitivity, evidenced by the lower values of blood glucose and insulin (52.2, 57.7, and 61.7%, respectively), as well as the HOMA-IR index.

**Figure 2:**
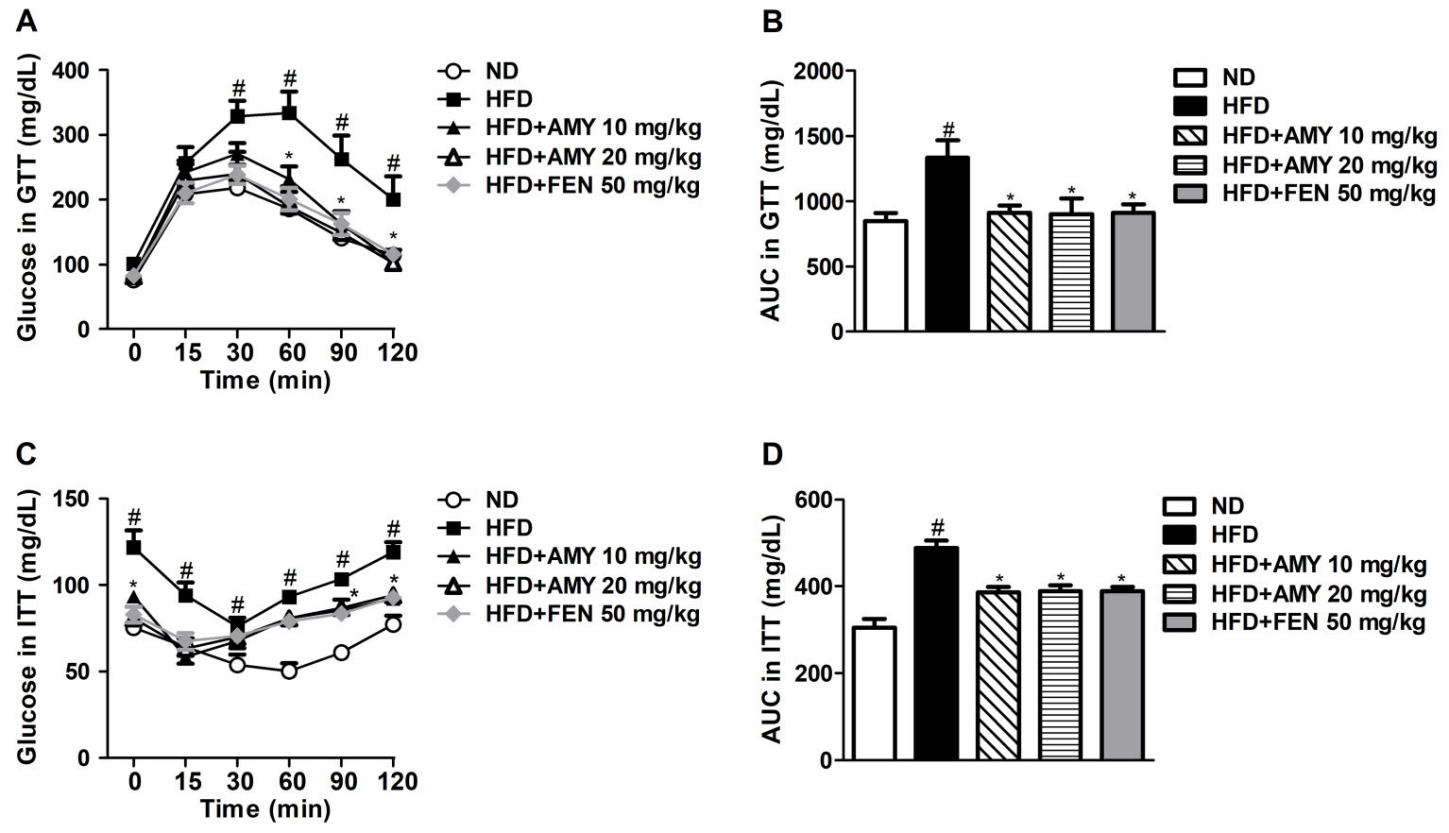
α,β-Amyrin (AMY) improved glucose tolerance and insulin resistance in HFD-fed mice. **A**, serum glucose level in glucose tolerance test (GTT); **B**, glucose area under the curve (AUC) during GTT; **C**, serum glucose in insulin tolerance test (ITT); **D**, glucose area under the curve (AUC) during ITT. Data are reported as means±SE (n=8). ^#^P<0.05 *vs* ND-fed animals; *P<0.05 *vs* HFD-fed animals (ANOVA followed by the Student*-*Newman*-*Keuls test or two-way ANOVA followed by the Bonferroni's test). ND: normal diet; HFD: high-fat diet; FEN: fenofibrate.

The GTT and ITT were performed to characterize whether AMY could improve hepatic insulin resistance. As shown in Figure 2, mice fed the HFD had significantly impaired glucose metabolism compared with ND-fed mice, indicating glucose intolerance. AMY and FEN treatments significantly reduced the increased glucose levels in GTT, suggesting they could improve HFD-induced glucose intolerance (Figure 2A and B). In the ITT, mice fed the HFD showed higher insulin levels compared to the ND group (Figure 2C and D), indicating lower insulin sensitivity. AMY and FEN treatments significantly reduced the insulin level in the ITT, suggesting these treatments can reduce HFD-induced insulin resistance (Figure 2C and D). However, in all these studied parameters, the effect of AMY was dose-independent.

### AMY improved serum lipid profiles

Hepatic lipid accumulation is a characteristic of NAFLD, and the effects of AMY on serum lipids were analyzed. As shown in Table 3, serum TG, TC, VLDL-c, and LDL-c were significantly increased in mice fed the HFD (by 23.4, 51.2, 24.9, and 115.5%, respectively) compared to mice fed the ND. AMY and FEN treatments markedly reduced the serum levels of TG, TC, VLDL-c, and LDL-c compared to the HFD control group. There was no statistical difference of HDL-c levels between groups. Significant increases in serum ALT and AST levels (70.0 and 290.8%, respectively) were detected in the HFD control, compared with the ND group. However, these increases were greatly decreased by treatment with AMY at 10 and 20 mg/kg (35.4, 34.4, 59.3, and 63.4%, respectively) or FEN (33.8 and 65.5%, respectively). Hepatic tissue levels of TG, TC, and glycogen were significantly lower in groups treated with AMY or FEN compared with the HFD group (Table 3).

**Table 3:** Effect of α,β-amyrin (AMY) and fenofibrate (FEN) treatments on serum and liver parameters in mice fed on high-fat diet (HFD) or normal diet (ND) for 15 weeks.

Parameters	ND	HFD	HFD+AMY (10 mg/kg)	HFD+AMY (20 mg/kg)	HFD+FEN (50 mg/kg)
ALT (U/L)	46.53±0.94	79.12±3.75^#^	51.08±1.53*	51.87±2.65*	52.37±2.36*
AST (U/L)	48.46±2.03	189.50±5.99^#^	77.05±4.19*	69.30±4.66*	65.34±4.29*
TG (mg/dL)	75.75±1.52	93.50±7.88^#^	60.00±3.53*	69.00±5.43*	71.17±7.70*
TC (mg/dL)	95.44±3.73	144.3±5.44^#^	125.2±10.03*	111.5±4.27*	93.12±4.30*
HDL-c (mg/dL)	71.00±2.14	73.17±2.30	74.00±2.30	69.17±3.66	67.96±2.78
VLDL-c (mg/dL)	14.55±0.52	18.17±1.43^#^	11.54±0.62*	12.30±0.62*	13.53±1.70*
LDL-c (mg/dL)	25.20±4.67	54.32±3.33^#^	43.32±2.73*	37.84±2.42*	28.48±5.50*
Liver weight (mg/g)	42.75±1.10	50.72±1.40^#^	36.33±1.26*	36.99±1.45*	41.53±1.84*
Liver TG (mg/g)	1.14±0.13	2.34±0.06^#^	0.98±0.10*	0.53±0.05*	0.72±0.02*
Liver TC (mg/g)	0.36±0.01	0.56±0.03^#^	0.44±0.01*	0.45±0.01*	0.48±0.01*
Hepatic glycogen (mg/g)	29.90±2.28	64.29 ±11.16^#^	21.31±3.82*	21.97±1.92*	24.06±2.14*

Data are reported as means±SE (n=8). ^#^P<0.05 *vs* ND-fed animals; *P<0.05 *vs* HFD-fed animals (one-way ANOVA followed by Student Newman Keul's multiple comparison *post hoc* test). ALT: alanine transaminase; AST: aspartate transaminase; TG: triglycerides; TC: total cholesterol; HDL-c: high-density lipoprotein cholesterol; VLDL: very-low-density lipoprotein; LDL-c: low-density lipoprotein cholesterol.

### Histological assessment of AMY protection against hepatosteatosis and steatohepatitis

**Figure 3:**
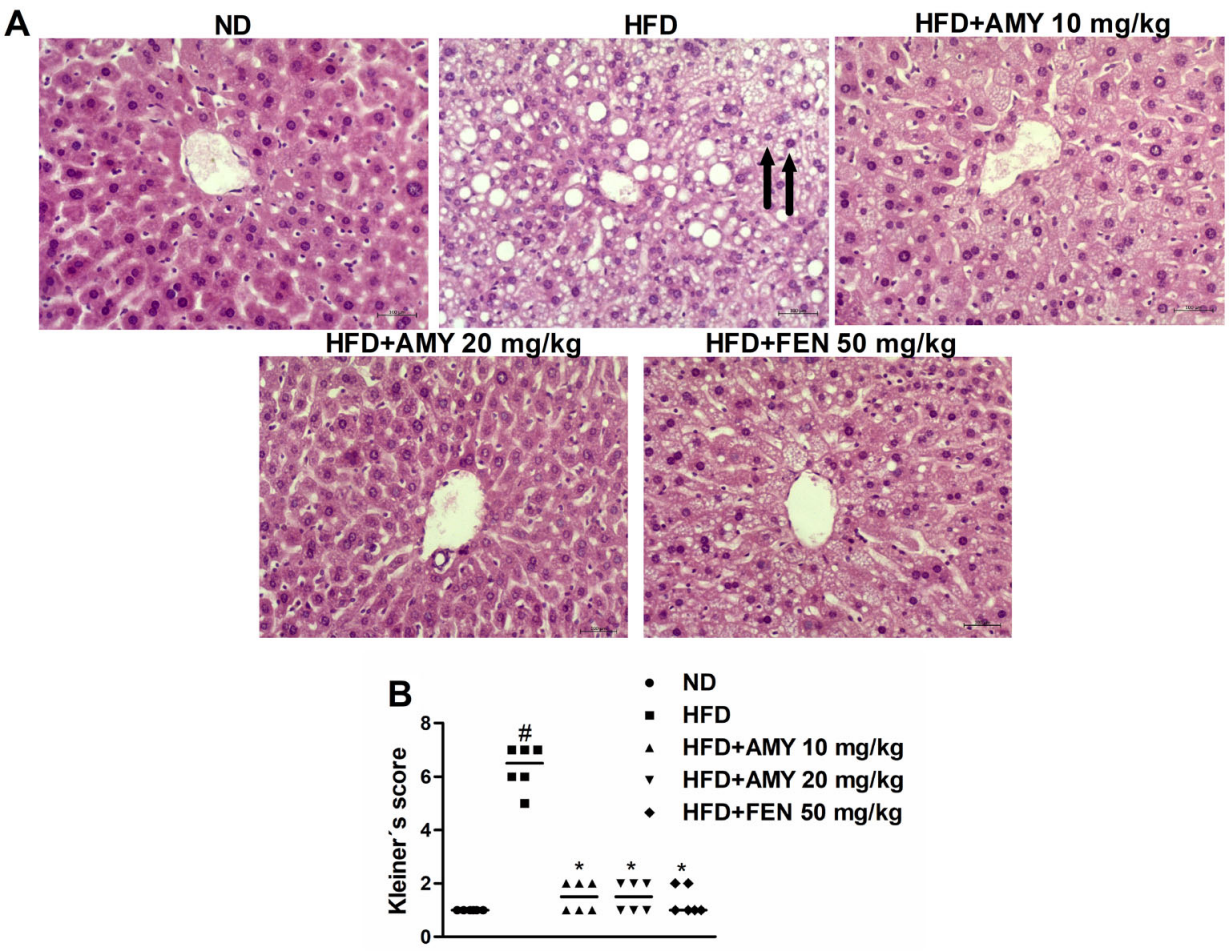
**A**, Representative liver sections (H&E stained) from ND-fed group, HFD-fed group, HFD+AMY (10 mg/kg), HFD + AMY (20 mg/kg), and HFD + FEN (50 mg/kg) (magnification: 200×, scale bar: 100 μm). Arrows indicate hepatocyte ballooning. **B**, The Kleiner's score of steatosis, lobular inflammation, and hepatocyte ballooning. Data are reported as median values (n=6). ^#^P<0.05 *vs* ND-fed animals; *P<0.05 *vs* HFD-fed animals (Kruskal-Wallis followed by Dunn's test). ND: normal diet; HFD: high-fat diet; AMY: α,β-amyrin; FEN: fenofibrate.

Characteristic histological features of NAFLD include steatosis, inflammation, hepatocellular ballooning, hepatocyte necrosis, and fibrosis (32). In the current study, liver sections from HFD-fed mice revealed the presence of steatosis, lobular inflammation, and ballooning with no evidence of cell necrosis and fibrosis compared to the parameters of ND-fed mice (Figure 3A). On the other hand, HFD-fed mice receiving AMY and FEN treatments showed significantly less steatosis and lobular inflammation and no ballooning. The respective Kleiner's score in HFD-fed mice livers were on the order of 6.5 (1-2) whereas these scores were markedly lower in animal groups treated with AMY at 10 mg/kg (1.5 (1-2)) and 20 mg/kg (1.5 ((1–2)), or FEN (1.0 (1–2)) (Figure 3B).

### AMY altered liver genes and proteins involved with lipid metabolism

**Figure 4:**
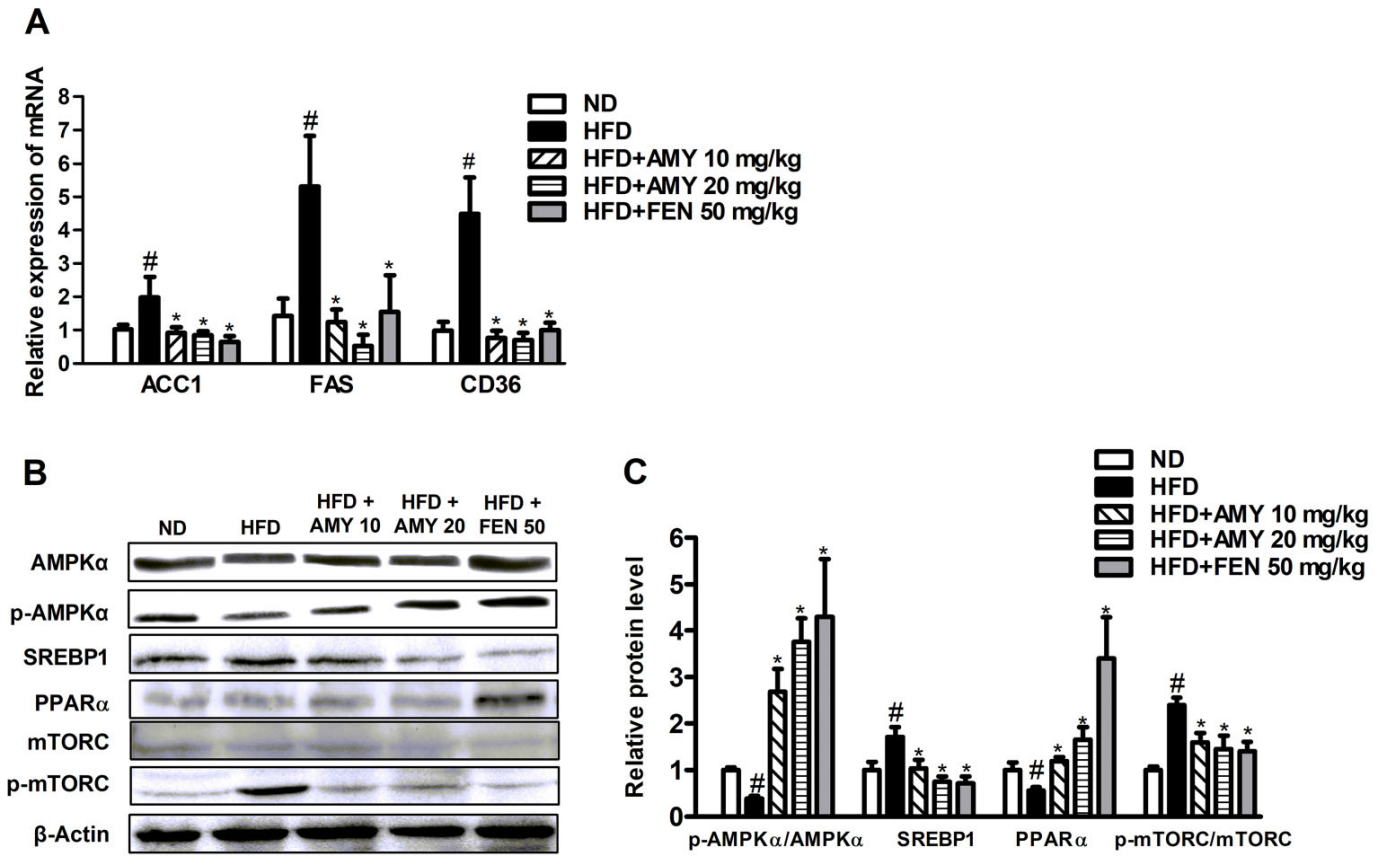
Effects of α,β-amyrin (AMY) on key molecules in liver lipid metabolism in HFD-induced nonalcoholic fatty liver disease in mice. **A**, RT-qPCR analyses of hepatic mRNA levels of genes involved in lipogenesis (ACC1 and FAS) and fatty acid uptake (CD36). **B** and **C**, Hepatic levels of proteins involved in energetic homeostasis (AMPK), lipogenesis (SREBP1 and mTORC), and beta oxidation (PPARα). Data are reported as means±SE (n=8). ^#^P<0.05 *vs* ND-fed animals; *P<0.05 *vs* HFD-fed animals (ANOVA followed by the Student-Newman*-*Keuls test). ND: normal diet; HFD: high-fat diet; FEN: fenofibrate.

Lipid accumulation in hepatocytes and high hepatic triglyceride content are the main features of NAFLD. As shown in Figure 4A, the mRNA levels of genes involved in lipogenesis (ACC1 and FAS) and fatty acid uptake (CD36) were up-regulated in liver tissues of HFD-fed mice compared with ND-fed mice. AMY and FEN treatments markedly decreased these levels, reflecting reduced lipogenesis and fatty acid uptake (Figure 4A). While the hepatic expression levels of mTORC1 and SERBP1 proteins were markedly elevated, a significant decrease in AMPK and PPARα levels was observed in the HFD group compared to the ND mice. In contrast, AMY and FEN treatments caused a significantly increase in hepatic levels of AMPK and PPARα, whereas the levels of SREBP1 and mTORC1 were markedly decreased compared to the HFD group (Figure 4B and C).

## Discussion

NAFLD is a global health problem among obese individuals (33). To mimic the pathological features of these NAFLD subjects, this study utilized the HFD-induced mouse model of NAFLD to investigate the beneficial effects of AMY.

The results of this study suggested that the triterpenoid AMY plays a pivotal role in modulating lipid metabolism in the liver. AMY treatment in HFD-fed mice is associated with significant decreases in visceral fat, liver weight, hepatosteatosis, NASH, and dyslipidemia. Histological observations further confirmed that AMY is effective in suppressing hepatosteotosis, hepatic inflammation (steatohepatitis), and hepatocytic ballooning (assessed by Kleiner's scoring). Although, HFD administration has been a widely used model to induce NAFLD in mice, one limitation of this model is that it does not demonstrate significant NAFLD progression (32), which may be the reason why liver sections from HFD controls did not evidence liver cell death or fibrosis, probably requiring much longer HFD feeding than 15 weeks. When we examined the potential of AMY to ameliorate HFD-induced glucose intolerance and insulin insensitivity, the respective GTT and ITT results showed improvement of HFD-induced insulin resistance, evidenced by a decrease in the HOMA-IR index. These beneficial effects of AMY against HFD-induced NAFLD suggested its usefulness for the prevention of nonalcoholic liver damage. To understand better the mechanism underlying these protective effects of AMY on hepatic steatosis, dyslipidemia, and insulin resistance, we analyzed the functioning of AMY on cell signaling mechanisms that regulate glucose and lipid metabolism.

In this study, consistent with an earlier observation (20), the protein expression of AMPKα was decreased in the liver tissue of HFD-fed mice, a factor with a large contribution to lipid accumulation in the NAFLD mice (19,34). AMY significantly and dose-independently stimulated AMPK activity and inhibited lipogenic genes such as fatty acid synthase (FAS) and ACC expression in the hepatic tissues, in a manner comparable to the effects of fenofibrate. This result indicated that AMY ameliorated abnormal lipid metabolism through the suppression of lipogenesis and the promotion of fatty acid oxidation via the upregulation of AMPK. The decreased lipid deposition in AMY-treated mouse liver tissue might also be in part due to impaired fat absorption from the gut, a result of AMPK upregulation-mediated decrease in the secretion of lipolytic enzymes.

It is well established that the HFD can induce hepatic steatosis in rodents through an increase in the expression of transcription factors like SREBP1c, a major activator of lipogenic genes and peroxisome proliferator-activated receptor alpha (PPARα), and the enzymes FAS and ACC in mouse livers (35). Liver AMPK activity mediates suppression of lipogenic gene expression (CD36, FAS, and ACC) by directly phosphorylating the master transcriptional regulators of lipogenesis SREBP1c (36,37). The cluster of differentiation 36 (CD 36) is one of the plasma membrane transporter proteins that facilitate the uptake of free fatty acids (FFAs), and excessive FFAs are taken by hepatocytes and converted into triglycerides in the form of intracellular lipid droplets, which is the hallmark of NAFLD (38). Besides CD 36, FAS, a rate-limiting enzyme that participates in the biosynthesis of fatty acids, and ACC, which plays a role in the glycolysis of glucose, also contribute to the hepatic accumulation of triglycerides. Thus, activating AMPK and/or inhibiting SREBP1 may be a therapeutic option for the control of NAFLD (36,38).

In this study, AMY significantly altered the expressions of SREBP1 and PPAR-α, and inhibited the lipogenic factors CD36, FAS, and ACC in hepatic tissues, indicating that AMY ameliorated NAFLD through the suppression of lipogenesis and the promotion of fatty acid oxidation. The ameliorating effect of AMY against NAFLD appeared to simulate fenofibrate, a known lipid-lowering drug used as a control, which is in fact a PPAR-α agonist that functions through the activation of lipoprotein lipase, and by inducing changes in the transcription of genes encoding enzymes involved in lipid and lipoprotein metabolism (39). In the liver, it is well established that the activation of both SREBP1 and mTORC1 transcriptions regulates *de novo* lipogenesis and biosynthesis of FFAs and TG, contributing to NAFLD pathogenesis (40). In the current study, the AMY treatment increased the AMPK activity in liver tissue and levels of SREBP1, and decreased mTORC1 activity, resulting in decreased lipogenesis and lipid accumulation. Thus, AMY treatment significantly blocked lipogenesis, acting as an inhibitor of mTORC1 signaling and SREBP1 transcription in livers of HFD-fed mice. Taken together, our results suggest that AMY effectively ameliorates intracellular lipid accumulation in liver cells, so AMY is a potential therapeutic agent for the prevention of fatty liver disease.

In conclusion, our data suggested that the plant triterpenoid AMY can ameliorate hepatosteatosis and steatohepatitis in mice with NAFLD induced by high-fat diet. The mechanism of reduced lipid accumulation in liver tissue of the AMY-treated HFD mice was likely the inhibition of lipogenesis, because of suppression of lipogenic genes and increase of fatty acid oxidation, involving the AMPK, mTORC1, and SREBP1 signaling pathways. Because NAFLD is becoming a public health problem of epidemic proportions, AMY may be a suitable candidate for drug development against NAFLD.
